# Climatic Associations of British Species Distributions Show Good Transferability in Time but Low Predictive Accuracy for Range Change

**DOI:** 10.1371/journal.pone.0040212

**Published:** 2012-07-05

**Authors:** Giovanni Rapacciuolo, David B. Roy, Simon Gillings, Richard Fox, Kevin Walker, Andy Purvis

**Affiliations:** 1 Division of Ecology and Evolution, Imperial College London, Ascot, Berkshire, United Kingdom; 2 Biological Records Centre, Centre for Ecology and Hydrology, Wallingford, Oxfordshire, United Kingdom; 3 Grantham Institute for Climate Change, Imperial College London, London, United Kingdom; 4 British Trust for Ornithology, Thetford, Norfolk, United Kingdom; 5 Butterfly Conservation, Wareham, Dorset, United Kingdom; 6 Botanical Society of the British Isles, Harrogate, North Yorkshire, United Kingdom; University of Western Australia, Australia

## Abstract

Conservation planners often wish to predict how species distributions will change in response to environmental changes. Species distribution models (SDMs) are the primary tool for making such predictions. Many methods are widely used; however, they all make simplifying assumptions, and predictions can therefore be subject to high uncertainty. With global change well underway, field records of observed range shifts are increasingly being used for testing SDM transferability. We used an unprecedented distribution dataset documenting recent range changes of British vascular plants, birds, and butterflies to test whether correlative SDMs based on climate change provide useful approximations of potential distribution shifts. We modelled past species distributions from climate using nine single techniques and a consensus approach, and projected the geographical extent of these models to a more recent time period based on climate change; we then compared model predictions with recent observed distributions in order to estimate the temporal transferability and prediction accuracy of our models. We also evaluated the relative effect of methodological and taxonomic variation on the performance of SDMs. Models showed good transferability in time when assessed using widespread metrics of accuracy. However, models had low accuracy to predict where occupancy status changed between time periods, especially for declining species. Model performance varied greatly among species within major taxa, but there was also considerable variation among modelling frameworks. Past climatic associations of British species distributions retain a high explanatory power when transferred to recent time – due to their accuracy to predict large areas retained by species – but fail to capture relevant predictors of change. We strongly emphasize the need for caution when using SDMs to predict shifts in species distributions: high explanatory power on temporally-independent records – as assessed using widespread metrics – need not indicate a model’s ability to predict the future.

## Introduction

Many species have responded to recent environmental change by shifting their distributions [1]–[3]. Predicting how distributions will change in the face of future environmental change is key to developing effective strategies for the conservation of biodiversity, ecosystems and the services they support [4], [5].

Correlative species distribution models (SDMs) are the main tools for predicting impacts of environmental change on species distributions [6]–[8]. SDMs typically correlate currently-observed species occurrence and environmental explanatory variables that reflect hypothesised constraints on species persistence, such as climate and land use [8]. By updating environmental predictors to match future environmental change scenarios and/or environments in different regions, these statistical models can be used to predict shifts in species distributions in time and/or space – assuming they are transferable to environmental domains that differ from those used to build the models [9].

SDMs have gained huge popularity owing to their potential for generating predictions of distribution shifts from any set of species occurrence records together with readily-available environmental measurements and future scenarios, as well as their ease of implementation. As a result, pressing conservation concerns at the national and continental scale have so far primarily relied on these data and methods [4], [10]–[14]. However, it is now widely acknowledged that predictions from SDMs are subject to uncertainties stemming from several limitations and over-simplistic assumptions [6]–[8], [15]. For example, these approaches do not directly model factors such as biotic interactions and dispersal limitations, which instead may be accounted for indirectly through spurious correlations with abiotic environmental variables [16], [17]; when transferred in time and/or space, the failure to model changes in species interactions (e.g., release from competitors) and evolutionary processes (e.g., local adaptation) can lead to misleading projections of shifts in species distributions [18], [19].

Whilst these correlative models overlook several fundamental ecological and evolutionary processes, they may still generate useful approximations of potential distribution shifts at the appropriate spatial scale in the instances where they successfully capture relevant predictor variables [6]. Unfortunately, assessing whether they do is notoriously difficult since their main aim is to predict events that are yet to occur [20]; most studies thus measure the transferability of their models using a subset or re-sampled set of the distribution records used to build the models, a limited approach that can greatly inflate estimates of predictive accuracy [20]. For this reason, an emerging approach for estimating the true transferability of SDMs has been to validate model predictions against independent field records documenting shifts in species distributions to novel time periods [20]–[26] and regions [27]–[31]. However, published accounts of such independent model validation have generally lacked methodological or taxonomic breadth. To obtain an exhaustive picture, transferability must be assessed along both methodological and taxonomic axes of variation. First, models built using the same data but different statistical frameworks generate different predictions [32]–[34], with discrepancies being magnified when transferring them in time [20], [24], [25]. Second, the accuracy of model predictions has also been found to vary greatly among taxa [25], [35]–[37], with some species lending themselves to more accurate prediction than others.

We test the temporal transferability of climate-based SDMs by drawing on an exceptionally-detailed dataset including distribution records for three of the best-studied sets of species in the world – the vascular plants, non-migratory butterflies and breeding birds of Great Britain – in two time periods, reflecting observed changes over a 20–40 year interval. For each species, we model distribution records as a function of climate in the first time period (calibration data) using ten of the most commonly-used species distribution modelling frameworks. We then project the calibrated model to the second time period, based on observed climate change, and compare projections with observed records (independent validation data) to derive reliable estimates of the prediction accuracy of the models built.

Using this approach, we assess whether simple correlative SDMs based solely on climate predictors – the environmental predictors for which we have the best understanding of likely future changes – can in some cases provide useful approximations of potential distribution shifts; and begin to describe the circumstances under which they may do so. Specifically, we ask three questions: (1) Are climate-based SDMs transferable in time? (2) Can they capture drivers of expansion and contraction of species geographic ranges? (3) What is the relative effect of methodological and taxonomic variation on prediction accuracy?

## Materials and Methods

### Species Distribution Data

We used distribution data for all vascular plants [38], [39], non-migratory butterflies [40], [41] and breeding birds [42], [43] of Great Britain at a 10 km grid square resolution. All species we modelled have distributions that extend beyond Great Britain across Europe; the effect on temporal transferability of calibrating models using local versus continental species distribution data remains an open question [44]. However, as well as offering unusually-detailed and high-quality distribution data, Great Britain is an island with its own separate history of environmental change; environmental drivers of distribution size and change in British populations are thus likely to differ somewhat from those of continental populations of the same species. For this reason, we only used records at the British extent to predict distribution change across Great Britain. For each group, we used occurrence records from two time periods (t_1_ and t_2_), corresponding to the periods of intensive recording effort leading to the publication of national distribution atlases (see Table 1). To avoid problems related to building models with small sample sizes [45], we ran all analyses excluding species with fewer than 20, 30, 40, or 50 occurrence records across the study area in either time period; since there were no qualitative differences in the results among these filters, we present the most inclusive set of results (i.e., excluding only species with fewer than 20 records). This filter led to the exclusion of most recently-introduced vascular plants (neophytes), which are known to have been under-recorded in t_1_ as a rule [38], [39] and which therefore do not lend themselves to reliable modelling. However, 185 neophytes were left in the final species set for greater statistical power; their removal did not affect the results qualitatively (0.002 and 0.003 increases in validation AUC overall and for plants alone, respectively; detailed results not presented). Although the absence of species from each 10 km grid square could not be definitively recorded during sampling, most grid squares surveyed in each period (i.e., 92–100% of Great Britain’s 10 km grid squares) were meticulously sampled, with high levels of duplicate recording and under-recorded areas being targeted by extra recording schemes. Thus, we assumed that each surveyed grid square in which a species was not recorded (i.e., non-detection) represented an absence. We acknowledge that sampling extent and intensity did vary among surveys and taxonomic groups; we later discuss the potential implications of this heterogeneity on results. The final dataset comprised presence-absence distribution data for 1587 vascular plant, 53 butterfly and 183 bird species in Great Britain (2808 10 km grid squares).

**Table 1:** Dates and sources of the distribution records used.

Group	t_1_ (calibration)		t_2_ (validation)		
	Dates	Source	Dates	Source	
Vascular Plants	1930–1969	Perring and Walters (1962) +later records	1987–1999	Preston *et al.* (2002)	
Butterflies	1970–1982	Heath *et al.* (1984)	1995–1999	Asher *et al.* (2001)	
Breeding Birds	1968–1972	Sharrock (1976)	1988–1991	Gibbons *et al.* (1993)	

### Environmental Data

Monthly values of temperature, precipitation and cloud cover for each year between 1930 and 1999 were obtained from the CRU ts2.1 [46] and the CRU 61–90 [47]; these were used to calculate mean values for nine climate variables – separately for each t_1_ and t_2_ period – that reflect hypothesised physiological constraints on species survival and growth. We conducted Spearman’s rank correlations between all pairs of climate variables and dropped three variables that were highly correlated with others (Spearman’s ρ>0.85) to reduce the risk of overfitting during model calibration. The final six climate variables included in the models were mean temperature of the coldest month (MTCO, °C), mean temperature of the warmest month (MTWA, °C), ratio of actual to potential evapotranspiration (APET, standard moisture index), potential sunshine (PSUN, minutes), total annual precipitation (TPRE, mm), and the difference between total winter precipitation and total summer precipitation (PREvar, mm).

We also considered including additional environmental predictors of ecological relevance to our models. First, although changes in land use have been identified as fundamental drivers of change for many British species [48]–[52], we were unable to account for them in our models – like most other published accounts of temporal transferability of SDMs [20], [21], [24], [25] – due to the lack of data documenting habitat use in the earlier t_1_ period; detailed digitised maps of land use for the whole of Britain are not available until the UK Land Cover Map in 1990 [53].

Second, topography and geology variables can also be fundamental determinants of current and potential distributions of species, so their inclusion in SDMs aimed at predicting distribution changes under environmental change should be considered [54]. However, their use in this context can be problematic if the species do not respond directly to these variables but rather to factors that are correlated with them [55]. In those cases, topography and geology variables themselves have no predictive power in new environmental domains; their inclusion is likely to increase the calibration accuracy of models at the expense of their transferability through time. To test for this effect, we built all SDMs using two alternative sets of predictors: (a) climate predictors only; (b) climate predictors plus two topography (median and standard deviation of elevation, m) and five geology (percentage cover of five substrate classes in each 10 km grid square: igneous and metamorphic; peat; sedimentary acid; sedimentary basic; and superficial) predictors. We then compared the performance between models built using each set of predictors (Table S1). Models including geology and topography predictors as well as climate had a higher accuracy than models with climate only according to most performance measures calculated, including both calibration and validation AUC; however, they had a lower mean correct classification rate for squares having changed occupancy status between time periods (CCR_changed_, our measure of the accuracy of models to capture relevant predictors of change; see Materials and Methods subsection “Can climate-based SDMs capture drivers of expansion and contraction of species geographic ranges?”). For this reason, we decided to leave both topography and geology variables out from our final models.

### Species Distribution Models

We modelled distribution data for each species in period t_1_ as a function of climate for the corresponding period using nine different modelling frameworks. Seven were presence-absence modelling techniques implemented in the BIOMOD package for R [56]. These included one classification method (classification tree analysis, CTA), three regression methods (generalised linear models, GLMs; generalised additive models, GAMs; and multivariate adaptive regression splines, MARS), and three machine-learning methods (artificial neural networks, ANNs; generalised boosted models, GBMs; and random forests, RFs). In addition, we also modelled occurrence records using two presence-only modelling techniques. These were maximum entropy (MaxEnt), implemented as a stand-alone application [57], and a rectilinear envelope analogous to BIOCLIM (surface range envelope, SRE), also implemented in the BIOMOD package for R [56]. Besides providing a useful comparison, presence-only techniques provide a test of whether it is reasonable to use non-detections as hypothesised absences or whether models should be built only using occurrences when recorded absences are missing. The nine modelling techniques employed are all commonly used to predict changes in species distributions [4], [12], [13] and have been found to generate contrasting predictions of change when modelling comparable data [21], [32], [58]. Although it is common knowledge that some of the modelling techniques we used (e.g., CTA, SRE) generally perform less well than others [32], [33], we believe that their transferability in time is not as well-established; therefore, we decided to include them in our analysis to test the hypothesis that simpler statistical models may have higher transferability in time than more complex ones. We chose different modelling parameters to optimise each statistical technique (see Supporting Information, Appendix S1). We used the nine species-climate associations identified in period t_1_ to generate predictions of each species’ geographic distribution in (a) period t_1_ (interpolation to the same climate used to build the models) and (b) period t_2_ (extrapolation to the climate experienced in the more recent period), based on observed climate for the corresponding periods. It is important to note that some of the techniques used differ in their method of projecting identified climatic requirements to geographical space: all presence-absence techniques generate predictions of probability of occurrence; MaxEnt generates various types of output, but for an intuitive comparison with presence-absence techniques we used its logistic output, an estimation of probability of occurrence; SRE returns a binary classification whereby each location falling within the range of climates identified by the presence locations becomes a presence, otherwise it becomes an absence. In addition to predictions from these nine single models, we calculated the mean probability of occurrence from all seven presence-absence modelling techniques (abbreviated Mn(PA)) as a simple but efficient consensus method for combining the output of different single-models [58]; this approach can reduce model-based uncertainty in predictions from SDMs [59]. To check that the results were not biased by the direction of modelling, we also carried out all analyses using the inverse approach, producing hindcasts in period t_1_ from models built in period t_2_.

### Are Climate-based SDMs Transferable in Time?

To quantify the transferability of SDMs in time, we measured the agreement between forecasts in period t_2_– as generated by each of the nine single-models built in period t_1_ plus the consensus method – and observed presence-absence for the corresponding period using three alternative measures of prediction accuracy [60]: (i) area under curve (AUC) of the receiver operating characteristic (ROC) function, (ii) sensitivity (i.e., proportion of correctly-predicted presences), and (iii) specificity (i.e., proportion of correctly-predicted absences). AUC is one of the most frequently-used measures of SDM performance as it removes the need to select a threshold to split continuous probabilities of occurrence into binary-transformed values, a process that is often viewed as subjective and misleading [61]. Swets [62] provided the following guidelines for interpreting AUC scores: 0.5≤ AUC <0.6 =  fail; 0.6≤ AUC <0.7 =  poor; 0.7≤ AUC <0.8 =  fair; 0.8≤ AUC <0.9 =  good; 0.9≤ AUC =  excellent; despite known limitations [63], [64], these are still widely-used, so we were interested in the conclusions reached based on them. To complement AUC scores, we calculated specificity and sensitivity for each model. This requires selecting an appropriate probability threshold to turn continuous probabilities of occurrence into binary presence-absence predictions. For each model, we calculated the sum of sensitivity and specificity on calibration data for 100 threshold values (in 0.01 increments), and selected the threshold that maximized this sum; this threshold has previously been found to perform well in comparisons with others [63]. Predicted probabilities of occurrence at time t_2_ above the selected threshold were converted to presences and those below to absences.

### Can Climate-based SDMs Capture Drivers of Expansion and Contraction of Species Geographic Ranges?

Quantifying the temporal transferability of SDMs by comparing the agreement between model predictions and observations for the predicted period using common metrics is not a sufficient test of whether models have actually captured relevant predictors of change. A single range-wide measure of prediction accuracy conflates accurately predicting species expansions and contractions to new areas with accurately predicting large parts of the distribution that have remained unchanged in time. Thus, to assess how well SDMs capture drivers of *change* in species distributions, we measured the agreement between observations and model predictions of each species’ (a) geographic range size in period t_2_, (b) overall change in geographic range size between time periods, and (c) grid square-level changes in occupancy status between time periods. By performing direct comparisons of observed records in each time period to derive measures of observed range change, we assumed the distribution data could be taken at face value, with no need to correct for sampling bias. While this is probably reasonable for British birds – for which similar analyses have already been carried out [21] – we acknowledge that sampling biases have been documented for British butterflies and plants, and various approaches have been applied for minimising those [49], [65]; we later discuss the potential implications of bias on our results.

We measured the agreement between observed and predicted range size in t_2_ and between observed and predicted overall change in range size across time periods using Spearman’s *ρ* statistic. To calculate the agreement between observed and predicted grid square-level changes in occupancy status, we divided binary forecasts into (a) grid squares that had either remained occupied or remained unoccupied between time periods and (b) grid squares that had changed occupancy status (from occupied to unoccupied or vice versa) between time periods. We then measured the correct classification rate (CCR; i.e., the sum of true positives and true negatives divided by the total number of locations) of grid squares in each of these two subsets for each modelling technique, to capture how well our models predict stable versus dynamic portions of each species’ distribution. To visualise model accuracy for expanding versus contracting species, we fitted generalised additive models (GAMs; using a cubic spline smoother with 4 degrees of freedom) of CCR of stable (CCR_stable_) and changed (CCR_changed_) grid squares as a function of observed proportional range change between time periods (i.e., (overall range change/range size in t_1_) x 100). These GAMs were fitted only to species experiencing a proportional change between −100% and +100% (i.e., 85% of all species), due to the large influence of the few species whose ranges more than doubled.

### What is the Relative Effect of Methodological and Taxonomic Variation on Prediction Accuracy?

We investigated the factors influencing the prediction accuracy of SDMs through linear mixed-effects (LME) models using the lme4 package in R [66]. We used five measures of accuracy (validation AUC, sensitivity, specificity, CCR_stable_, and CCR_changed_) in turn as the response and modelled each as a function of the following random effects: modelling framework (n = 10), major taxonomic group (i.e., plants, butterflies or birds; n = 3) and species (n = 1823). For each model, we calculated the ratio between the variance explained by each random effect and null variance, in order to quantify the amount of variation in prediction accuracy attributable to each random effect.

## Results

Due to similarities between model forecasts and hindcasts, we direct our attention to the analysis of forecasts, referring to hindcasts only when qualitatively different; the results of hindcasts are reported in full in the Supporting Information. The species distribution models (SDMs) built using data in t_1_ had an AUC  = 0.85±0.12 (mean±s.d.), indicating good fit on calibration data overall according to the Swets criterion [62].

### Are Climate-based SDMs Transferable in Time?

**Figure 1:**
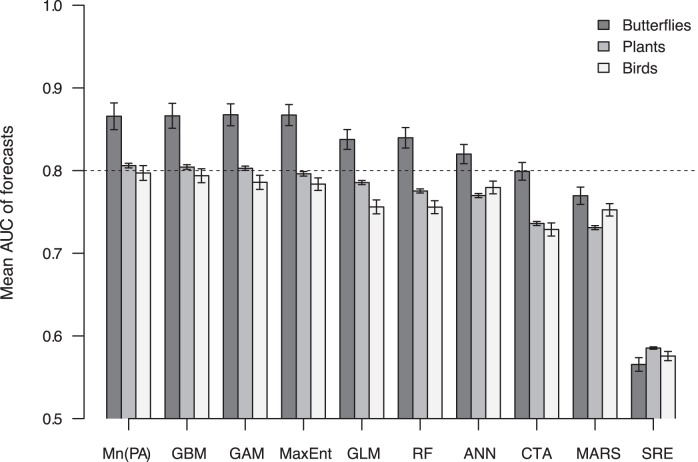
Accuracy of model forecasts. The accuracy of forecasts generated by each modelling framework was measured by mean AUC and reported for each major taxonomic group. Error bars represent ±1 standard error of the mean. The dashed line indicates the rule-of-thumb for good predictions (AUC = 0.8). Abbreviations: ANN  =  artificial neural networks, CTA  =  classification tree analysis, GAM  =  generalised additive models, GBM  =  generalised boosted models, GLM  =  generalised linear models, MARS  =  Multivariate adaptive regression splines, MaxEnt  =  maximum entropy models, Mn(PA)  =  prediction mean from all presence-absence modelling frameworks, RF  =  random forests, SRE  =  surface range envelopes.

**Figure 2:**
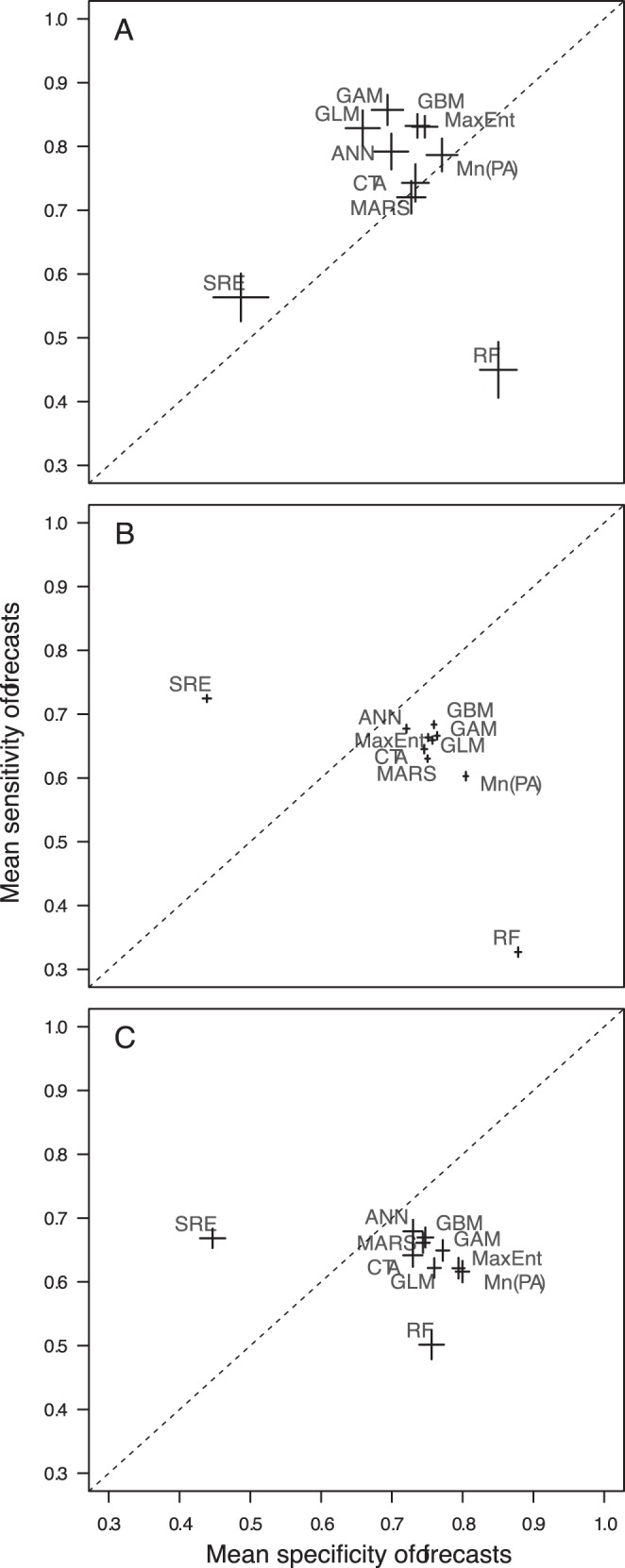
Mean sensitivity versus mean specificity of model forecasts. Mean sensitivity and specificity of forecasts generated by each modelling framework for (A) butterflies, (B) plants, (C) birds. Error bars represent ±1 standard error of the mean. The dotted line indicates the condition where mean sensitivity  =  mean specificity.

The overall transferability of SDMs in time was fair (mean AUC±s.d.  = 0.76±0.12; sensitivity  = 0.63±0.26; specificity  = 0.74±0.19), but varied among modelling frameworks. The consensus method Mn(PA) produced the highest validation AUC values (Figure 1), generating good to excellent forecasts (AUC ≥0.80) for 60% of the 1823 species modelled. Among single-models, three presence-absence techniques – generalised boosted models (GBMs), generalised additive models (GAMs), generalised linear models (GLMs) – and one presence-only technique – maximum entropy (MaxEnt) – had the highest prediction accuracies, although their relative rank varied between forecasts and hindcasts (Figure 1 and Figure S1). When assessed using sensitivity and specificity (Figure 2), GBMs and GAMs had the best balance between the correct prediction of presences and absences; in contrast, random forests (RFs) were highly biased towards the correct prediction of absences and surface range envelopes (SREs) were highly biased the other way. These differences were reflected in the proportion of species for which each technique was most accurate according to alternative metrics: SREs generated the most accurate forecasts for 36% of all species when assessed by sensitivity whilst RFs generated the most accurate forecasts for 69% of all species when assessed by specificity (Table 2). Despite these differences, every SDM framework used performed best for some species modelled (Table 2 and Table S2), indicating that any one of them might be the most useful in at least some cases. Temporal transferability also varied among taxonomic groups (Figure 1 and Figure 2); it was highest for butterflies, with 66% of butterfly models predicting recent distributions with good to excellent accuracy against 43% and 41% for plants and birds, respectively. Hindcasts showed slightly different results, with bird distributions in t_1_ being predicted almost as well as those of butterflies and considerably better than those of plants (Figure S1 and Figure S2).

**Table 2:** Number of species for which each modelling framework generated the most accurate forecasts.

Number (and proportion) of best-predicted species					
	AUC	Sensitivity	Specificity	CCR_stable_	CCR_changed_
Mn(PA)	393 (0.216)	79 (0.043)	203 (0.111)	117 (0.064)	84 (0.046)
RF	25 (0.014)	27 (0.015)	1254 (0.688)	1090 (0.600)	234 (0.128)
GBM	234 (0.128)	168 (0.092)	39 (0.021)	77 (0.042)	117 (0.064)
MaxEnt	311 (0.171)	159 (0.087)	92 (0.050)	46 (0.025)	174 (0.095)
GAM	397 (0.218)	131 (0.072)	53 (0.029)	99 (0.054)	98 (0.054)
GLM	138 (0.076)	125 (0.069)	30 (0.016)	46 (0.025)	119 (0.065)
ANN	256 (0.140)	316 (0.173)	102 (0.056)	125 (0.069)	210 (0.115)
MARS	61 (0.033)	113 (0.062)	36 (0.020)	55 (0.030)	103 (0.057)
CTA	6 (0.003)	226 (0.124)	63 (0.035)	105 (0.058)	179 (0.098)
SRE	3 (0.002)	654 (0.359)	6 (0.003)	101 (0.055)	717 (0.393)

Prediction accuracy was measured for each species by AUC, sensitivity, and specificity of the entire range in t_2_, as well as the correct classification rate of grid squares that have remained occupied or unoccupied (CCR_stable_) and the correct classification rate of grid squares that have changed occupancy status between time periods (CCR_changed_). Values represent the total number (and proportion of the total sample) of species for which each technique performed best. Proportions may exceed 100% of the sample as several species were equally well-predicted by more than one technique.

### Can Climate-based SDMs Capture Drivers of Expansion and Contraction of Species Geographic Ranges?

Predicted and observed range sizes in t_2_ were highly correlated for each modelling framework and taxonomic group (Table 3); overall, SDMs tended to overpredict range size, with a median percentage overprediction of 7.44%. Among modelling frameworks, RFs showed the highest correlation; contrary to the overall trend, RFs tended to systematically under-predict range size (median percentage underprediction of −5.02%), again highlighting their high specificity but low sensitivity. Among taxonomic groups, butterflies showed the highest correlation.

**Table 3:** Correlation coefficients of observed versus predicted range size and range change for model forecasts.

	Butterflies		Plants		Birds	
	Range size	Range change	Range size	Range change	Range size	Range change
Mn(PA)	0.76^***^	−0.10	0.68^***^	−0.01	0.72^***^	0.36^***^
RF	0.96^***^	0.43^**^	0.85^***^	−0.02	0.95^***^	0.38^***^
GBM	0.87^***^	−0.23	0.63^***^	−0.04	0.75^***^	0.45^***^
MaxEnt	0.82^***^	−0.23	0.52^***^	0.01	0.72^***^	0.41^***^
GAM	0.84^***^	−0.11	0.58^***^	−0.03	0.80^***^	0.38^***^
GLM	0.80^***^	−0.11	0.60^***^	−0.02	0.80^***^	0.36^***^
ANN	0.65^***^	−0.19	0.45^***^	−0.03	0.44^***^	0.35^***^
MARS	0.72^***^	−0.39	0.62^***^	−0.04	0.81^***^	0.43^***^
CTA	0.87^***^	0.02	0.58^***^	−0.07^**^	0.76^***^	0.40^***^
SRE	0.90^***^	0.13	0.89^***^	0.21^***^	0.90^***^	0.35^***^

Reported values are the Spearman’s ρ coefficients of observed versus predicted range size in t_2_ (range size column) and observed versus predicted change in range size between time periods (range change column) for each modelling framework and major taxonomic group modelled. Stars indicate the significance level of correlations: ^*^  =  p<0.05; ^**^  =  p<0.01; ^***^  =  p<0.001.

Spearman’s ρ of predicted versus observed total change in range size between time periods were low overall, although higher for hindcasts (Table S3) than forecasts (Table 3). Species ranges were predicted to increase by a median 7.44% more than was observed; this difference was greater for species with contracting rather than expanding ranges (median overprediction 11.62% and 5.67%, respectively). Differences among taxa and frameworks were influenced by the direction of modelling (compare Table 3 and Table S3).

**Figure 3:**
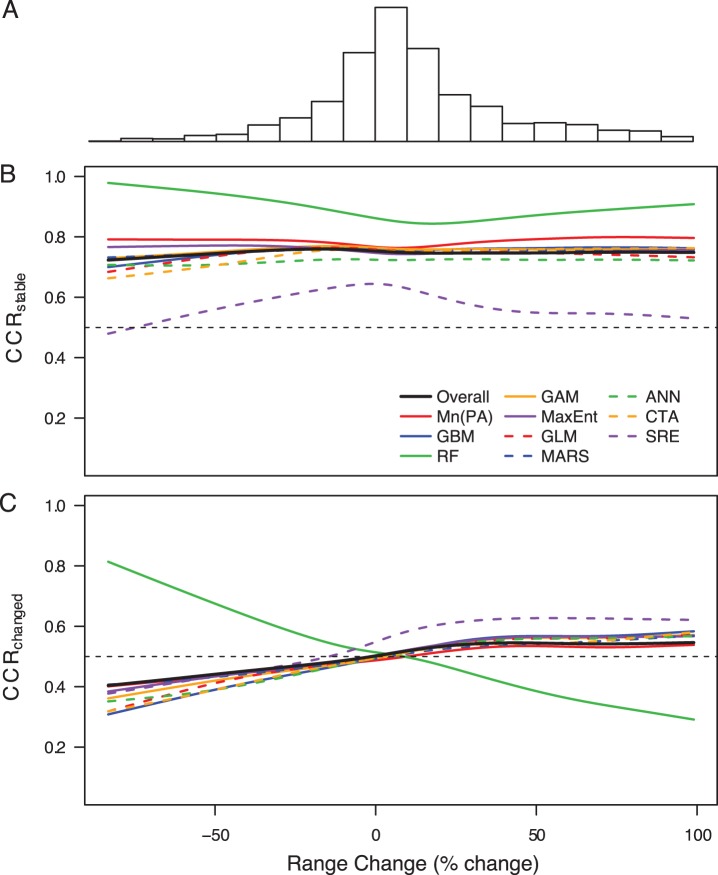
Accuracy of forecasted changes in species occupancy. Accuracy of predicted changes in occupancy between t_1_ and t_2_ as a function of species’ observed proportional range change between t_1_ and t_2_: (A) histogram of the frequency of species’ proportional range change values; (B) correct classification rate across stable grid squares (i.e., those that have remained either occupied or unoccupied between time periods; CCR_stable_) as a function of observed proportional range change, overall and for each modelling technique; (C) correct classification rate across changed grid squares (i.e., those that have changed occupancy status between time periods; CCR_changed_) as a function of observed proportional range change, overall and for each modelling technique. Functions were fitted using generalised additive models (GAM; using a cubic spline smoother with 4 degrees of freedom). This analysis was limited to species experiencing a proportional change between −100% and +100% (i.e., 85% of all species), due to the very high influence of the few species whose range more than doubled. The dashed line in panels (B) and (C) represents the value of CCR expected from a random guess (i.e., CCR  = 0.5).

The correct classification rate of grid squares that remained occupied or remained unoccupied (CCR_stable_) was fairly high (mean±s.d.  = 0.75±0.15), and did not covary with species’ observed proportional change in range size (Figure 3B). In contrast, the CCR of grid squares whose occupancy status changed between time periods (CCR_changed_) was very low overall (0.51±0.14; guessing randomly would be expected to produce a mean of 0.5), with range expansions being slightly better predicted than range contractions (0.55±0.15 and 0.48±0.12, respectively; Figure 3C). RFs showed an unusual trend compared to other frameworks: they had by far the highest CCR_stable_ (0.88±0.10; Figure 3B) and were the only framework to provide more accurate predictions for contracting than expanding species (Figure 3C). When hindcasting, the discrepancy between CCR_stable_ and CCR_changed_ was even larger; however, there was no clear difference between the prediction accuracy of expanding and contracting species (Fig. S3).

### What is the Relative Effect of Methodological and Taxonomic Variation on Prediction Accuracy?

Most variation in the prediction accuracy of SDMs – as measured by AUC, sensitivity, CCR_stable_, CCR_changed_ – was among species within a higher taxon, whilst the choice of modelling framework was as important a factor in explaining variation in specificity (Table 4 and Table S4). The effect of major taxonomic group on the accuracy of forecasts was relatively small.

**Table 4:** Relative effect of taxonomic and methodological variation on accuracy of forecasts.

	AUC	Sensitivity	Specificity	CCR_stable_	CCR_changed_
Species	46.26	36.53	25.90	35.50	33.62
Technique	29.05	14.73	32.33	19.05	4.24
Group	5.59	4.76	0.15	0.44	17.23
Residual	19.11	43.99	41.62	45.01	44.91

The values reported are the results of a variance components analysis of the linear mixed-effects (LME) models investigating the factors affecting the accuracy of forecasts. AUC, sensitivity, specificity of the entire range, as well as the correct classification rate of grid squares that have remained occupied or unoccupied (CCR_stable_), and the correct classification rate of grid squares that have changed occupancy status between time periods (CCR_changed_) were modelled as a function of the following random effects: modelling framework (n = 10), major taxonomic group (n = 3) and species (n = 1823). The ratio between the variance explained by each random effect and null variance (expressed as a percentage) is reported for each random effect in each model.

## Discussion

When assessed using widespread measures of performance such as the AUC, sensitivity and specificity, climate-based species distribution models (SDMs) show good transferability in time for many species and techniques. Our estimates of temporal transferability across all taxa and methods are comparable with those reported previously [20], [24]–[26]. However, predictions of changes in occupancy status between time periods as a function of climate change were little or no better than random for most species, regardless of the modelling framework used; models were particularly poor at predicting species range contractions, a worrying prospect in the context of forecasting environmental change impacts on species of conservation concern. There are many widely-acknowledged obstacles to the accurate prediction of shifts in species distributions in time [6], [8]: these include the lack of species-environment equilibrium [67]; dispersal limitations [68]; the failure to account for biotic interactions, phenotypic plasticity, and evolutionary changes [6], [69]; and the incidence of novel environments outside the range of conditions used to calibrate the models [9]. Together with these, three key factors appear likely to underlie the combination of reasonable *explanatory* power on independent data – as indicated by widespread metrics – but low *predictive* accuracy between time periods – as shown by the failure to predict changes in occupancy – of our models.

First, the two sets of distribution records we used for each species cannot be considered to be truly independent from each other as they were collected over the same geographical area within relatively short time intervals (i.e., 20 to 40 years). Overall, species range size was highly correlated between time periods (Spearman’s *ρ* = 0.93, p<0.001), with an average 87% of grid squares maintaining the same occupancy status; similarly, all climatic variables were also highly correlated between time periods (*ρ*>0.85, p<0.001 for all variables). As a result, models providing a good fit to early distribution records can be expected to return a reasonable fit to more recent records (and vice versa), regardless of whether relevant predictors of range shift have actually been captured. Previous studies have warned against taking strong model performance on calibration data to indicate high predictive accuracy to a different time period [20], [24]–[26]; our results indicate that strong model performance in a different time period, as measured by widespread metrics, may not indicate high predictive accuracy either.

Second, our models’ lack of power to predict observed changes in occupancy suggests that they are missing information on fundamental variables that drove those changes. Whilst climate change has undoubtedly had a significant effect, another major driver of change in species distributions in Britain over the second half of the 20^th^ century is thought to be habitat change, both degradation and fragmentation [70]. Lack of suitable habitat has constrained the ability of some British species to respond to changes in climate [49], [51], [52]; thus, models based exclusively on climate can generate misleading predictions of change [49], [51], [52]. Although data on land use exist for recent years (corresponding to time t_2_ of our distribution datasets [53]), we were unable to include variables describing habitat change in our models because data documenting land use in the earlier t_1_ period was not available; an issue which applies to most tests of temporal transferability of SDMs (but see [26]). However, climate and habitat variables affect species distributions at different spatial scales [6], [71], and climate is considered to be the main constraint at the scale of our study [71], [72]. Furthermore, the distribution of many habitat variables, such as forest and grassland cover, are themselves directly affected by climate [72]. Thus, the absence of habitat predictors from our models is unlikely to be solely responsible for their low predictive accuracy. Despite this, we acknowledge that grid square-level climatic averages ignore the high degree of environmental heterogeneity within each grid square; so long as patches of suitable environment remain within a grid square, species may persist even if that square’s climatic average says otherwise. Similarly, if suitable habitat disappears from a grid square, the species may be lost from it even if the average climate remains suitable. Consequently, the lack of habitat variables in our models may underlie their worse prediction of range contractions than of expansions. Although recent climate warming can be expected to increase the distributions of many species in Britain [49], [51], [52], some have in fact declined as a result of habitat change (e.g., non-migratory butterflies reaching their northern limits in Britain [49], [52]); predictions of shifts for these species based solely on climate are thus likely to be random, if not worse.

Finally, by assuming the non-detection of a species to indicate absence from a given grid cell, we introduced an extra level of error into our models. This error depends on the probability of false absence given imperfect detection (i.e., the probability that a species was present but remained undetected in a given grid cell [73]): the higher this probability, the higher the risk of incorrectly quantifying species-climate relationships [73]. The incidence of false absences is likely to vary among the three groups of species we modelled. A sustained effort to exhaustively target under-recorded areas plus a high level of duplicate recording for each 10 km grid square during both British breeding bird surveys imply a fairly low probability of false absence [43]. On the other hand, distribution records for British butterflies and plants are known to be biased – with significant differences in coverage and sampling effort between time periods [49], [65] – and are likely to suffer from a higher incidence of false absences, especially in t_1_. Although differences in the precision of distribution records may have contributed to the variation in prediction accuracy among the three groups, the low correlation we found between predicted and observed range changes for birds suggests that the low predictive power of our models cannot primarily be attributed to data quality.

The incidence of false absences is also likely to vary among surveys, with under-recording in t_1_ compared to t_2_ surveys having been documented for British butterflies and plants [49], [65]. Under-recording in t_1_ leads to overestimation of range expansions [74]. On the other hand, any inferred range contractions are likely to be robust. We would expect our models to have a high accuracy to predict false range expansions, given that these do not require models to predict change in time but simply to interpolate existing distributions based on detections in t_1_; conversely, range contractions can only be predicted by models capturing fundamental predictors of change. Due to the low accuracy of our models to predict range expansions and their even lower accuracy to predict range contractions, we conclude that differences in sampling effort between time periods cannot alone explain the low predictive power of our models; however, they may be responsible for some of the differences between forecasts and hindcasts.

Our analysis generated two key sets of results in the context of improving SDMs as predictive tools. First, differences among species were the most important determinant of variability in the temporal transferability of SDMs, as also reported recently for vascular plants in California [25]. These findings mirror those of earlier studies of variation in model performance based on distribution records from a single time period [75], [76], and suggest that a priority now is to identify the ecological context of species whose changes in distribution can be predicted accurately using existing techniques and widely-available environmental data. Differences in prediction accuracy among species are likely to be determined by a complex of ecological factors, including their intrinsic biology [25], [35]–[37], their history of dispersal [23], [77] and the identity and behaviour of their interacting species [69], [78], [79]. Encouragingly, there is some reason to believe that SDMs may be particularly useful for species of commercial importance (e.g., plantation trees [80]) – for which the above ecological factors are well-known and/or controlled – and, thus, species for which we are likely to require predictions of change very soon. In addition to identifying the sources of variation in prediction accuracy among models of different species, spatial analyses of per-site prediction successes and failures – aimed at identifying those locations in which models have high predictive accuracy across species – will also be important for a comprehensive understanding of the context in which SDMs may be useful.

Second, we identified some modelling frameworks to be more accurate than others overall, although none outperformed all others across all aspects. Whilst the consensus method we used provided the best predictions under AUC assessment – seemingly confirming its potential for reducing model-based uncertainty in SDM predictions [58], [59] – its accuracy to predict changes in occupancy was lower than most single models. As a result, we advocate great care when selecting the ensemble of models from which to derive consensus predictions; as previously discussed by Araújo *et al.* [21], models should be chosen based on aspects of their individual performance pertinent to the research question being addressed, and not on the assumption that more models are better. Furthermore, our results suggest that the presence-only modelling framework maximum entropy (MaxEnt) can be used to generate predictions of change of similar accuracy to those generated by the best-performing presence-absence frameworks – generalised boosted models (GBMs), generalised additive models (GAMs), and generalised linear models (GLMs).

In conclusion, some of the modelling tools already in place seem able to make use of presence-only or presence-absence distribution data and climate predictors to generate SDMs that are transferable in time for many species, when assessed using widespread measures of performance. However, a more in-depth assessment indicates they are inadequate at predicting changes in occupancy between time periods for most species, stressing the need to account for additional drivers and mechanisms of change. Despite their issues, SDMs still represent the most plausible framework for generating urgent predictions of the fate of biodiversity during a period of rapid environmental change. Observed shifts in species distributions provide invaluable opportunities for testing their predictions. Nevertheless, we strongly emphasize the need for caution: assessment of performance should not focus on the ability of models to predict large areas retained by species through time but rather on their success to capture relevant drivers of change.

## Supporting Information

Figure S1 **Accuracy of model hindcasts.** The accuracy of hindcasts generated by each modelling framework was measured by mean AUC and reported for each major taxonomic group. Error bars represent ±1 standard error of the mean. The dashed line indicates the rule-of-thumb for good predictions (AUC = 0.8). Abbreviations: ANN  =  artificial neural networks, CTA  =  classification tree analysis, GAM  =  generalised additive models, GBM  =  generalised boosted models, GLM  =  generalised linear models, MARS  =  Multivariate adaptive regression splines, MaxEnt  =  maximum entropy models, Mn(PA)  =  prediction mean from all presence-absence modelling frameworks, RF  =  random forests, SRE  =  surface range envelopes.
(EPS)

Figure S2 **Mean sensitivity versus mean specificity of model hindcasts.** Mean sensitivity and specificity of hindcasts generated by each modelling framework for (A) butterflies, (B) plants, (C) birds. Error bars represent ±1 standard error of the mean. The dotted line indicates the condition where mean sensitivity  =  mean specificity.
(EPS)

Figure S3 **Accuracy of hindcasted changes in species occupancy**. Accuracy of predicted changes in occupancy between t_2_ and t_1_ as a function of species’ observed proportional range change between t_2_ and t_1_: (A) histogram of the frequency of species’ proportional range change values; (B) correct classification rate across stable grid squares (i.e., those that have remained either occupied or unoccupied between time periods; CCR_stable_) as a function of observed proportional range change, overall and for each modelling technique; (C) correct classification rate across changed grid squares (i.e., those that have changed occupancy status between time periods; CCR_changed_) as a function of observed proportional range change, overall and for each modelling technique. Functions were fitted using generalised additive models (GAM; using a cubic spline smoother with 4 degrees of freedom). This analysis was limited to species experiencing a proportional change between −100% and +100% (i.e., 85% of all species), due to the very high influence of the few species whose range more than doubled. The dashed line in panels (B) and (C) represents the value of CCR expected from a random guess (i.e., CCR  = 0.5).
(EPS)

Table S1 **Effect of predictor set on the accuracy of model forecasts and hindcasts.**
(DOCX)

Table S2 **Number of species for which each modelling framework generated the most accurate hindcasts.**
(DOCX)

Table S3 **Correlation coefficients of observed versus predicted range size and range change for model hindcasts.**
(DOCX)

Table S4 **Relative effect of taxonomic and methodological variation on accuracy of hindcasts.**
(DOCX)

Appendix S1 **Description and optimisation of the modelling techniques used.**
(DOCX)
