# Supplementary material for: Climatic Associations of British Species Distributions Show Good Transferability in Time but Low Predictive Accuracy for Range Change
Source: PLoS One. 2012 Jul 5;7(7):e40212. doi: 10.1371/journal.pone.0040212 (PMC3390350; doi:10.1371/journal.pone.0040212)
Supplement: Table S1 — Effect of predictor set on the accuracy of model forecasts and hindcasts. (DOCX) [file pone.0040212.s004.docx]

**Table S1. Effect of predictor set on accuracy of model forecasts and hindcasts**

|  | **Calibration AUC** | **Validation AUC** | **Validation Sensitivity** | **Validation**  **Specificity** | **CCR_stable_** | **CCR_changed_** |
| --- | --- | --- | --- | --- | --- | --- |
| *Forecasts* |  |  |  |  |  |  |
| Climate | 0.85 ± 0.12 | 0.76 ± 0.12 | 0.63 ± 0.26 | 0.74 ± 0.19 | 0.75 ± 0.15 | 0.51 ± 0.14 |
| Climate +  Geology + Topography | 0.86 ± 0.12 | 0.79 ± 0.12 | 0.67 ± 0.24 | 0.75 ± 0.18 | 0.79 ± 0.14 | 0.48 ± 0.14 |
| *Hindcasts* |  |  |  |  |  |  |
| Climate | 0.86 ± 0.12 | 0.76 ± 0.12 | 0.61 ± 0.27 | 0.72 ± 0.23 | 0.79 ± 0.16 | 0.46 ± 0.16 |
| Climate +  Geology + Topography | 0.87 ± 0.11 | 0.78 ± 0.12 | 0.64 ± 0.25 | 0.74 ± 0.21 | 0.81 ± 0.15 | 0.43 ± 0.13 |

Values correspond to mean ± s.d. prediction accuracy across all models (i.e., all modelling frameworks for all species, n = 18,230) for each modelling direction (forecasts vs hindcasts) for each of the two sets of predictors considered (climate vs climate + geology + topography). Prediction accuracy was measured by mean AUC (both during calibration and validation), mean validation sensitivity and mean validation specificity of the entire range in t_1_, as well as the correct classification rate of grid squares that have remained occupied or unoccupied (CCR_stable_) and the correct classification rate of grid squares that have changed occupancy status between time periods (CCR_changed_).
