# Supplementary material for: Climatic Associations of British Species Distributions Show Good Transferability in Time but Low Predictive Accuracy for Range Change
Source: PLoS One. 2012 Jul 5;7(7):e40212. doi: 10.1371/journal.pone.0040212 (PMC3390350; doi:10.1371/journal.pone.0040212)
Supplement: Table S2 — Number of species for which each modelling framework generated the most accurate hindcasts. (DOCX) [file pone.0040212.s005.docx]

**Table S2. Number of species for which each modelling framework generated the most accurate hindcasts.**

| **Number (and proportion) of best-predicted species** | | | | | |
| --- | --- | --- | --- | --- | --- |
|  | **AUC** | **Sensitivity** | **Specificity** | **CCR_stable_** | **CCR_changed_** |
| Mn(PA) | 344 (0.189) | 51 (0.028) | 223 (0.122) | 178 (0.098) | 153 (0.084) |
| RF | 27 (0.015) | 197 (0.108) | 1078 (0.591) | 1017 (0.558) | 622 (0.341) |
| GBM | 136 (0.075) | 71 (0.039) | 84 (0.046) | 90 (0.049) | 107 (0.059) |
| MaxEnt | 357 (0.196) | 109 (0.060) | 101 (0.055) | 86 (0.047) | 133 (0.073) |
| GAM | 382 (0.210) | 132 (0.072) | 63 (0.035) | 101 (0.055) | 99 (0.054) |
| GLM | 262 (0.144) | 149 (0.082) | 64 (0.035) | 88 (0.048) | 103 (0.057) |
| ANN | 193 (0.106) | 465 (0.255) | 81 (0.044) | 139 (0.076) | 197 (0.108) |
| MARS | 117 (0.064) | 79 (0.043) | 101 (0.055) | 96 (0.053) | 110 (0.060) |
| CTA | 5 (0.003) | 122 (0.067) | 118 (0.065) | 97 (0.053) | 188 (0.103) |
| SRE | 0 (0.000) | 576 (0.316) | 4 (0.002) | 12 (0.007) | 330 (0.181) |

Prediction accuracy was measured by AUC, sensitivity, and specificity of the entire range in t_1_, as well as the correct classification rate of grid squares that have remained occupied or unoccupied (CCR_stable_) and the correct classification rate of grid squares that have changed occupancy status between time periods (CCR_changed_). Values represent the total number (and proportion of the total sample) of species for which each technique performed best. Proportions may exceed 100% of the sample as several species were equally well-predicted by more than one technique.
