# Supplementary material for: Climatic Associations of British Species Distributions Show Good Transferability in Time but Low Predictive Accuracy for Range Change
Source: PLoS One. 2012 Jul 5;7(7):e40212. doi: 10.1371/journal.pone.0040212 (PMC3390350; doi:10.1371/journal.pone.0040212)
Supplement: Table S3 — Correlation coefficients of observed versus predicted range size and range change for model hindcasts. (DOCX) [file pone.0040212.s006.docx]

**Table S3. Correlation coefficients of observed versus predicted range size and range change for model hindcasts.**

|  | **Butterflies** | | **Plants** | | **Birds** | |
| --- | --- | --- | --- | --- | --- | --- |
|  | Range size | Range change | Range size | Range change | Range size | Range change |
| Mn(PA) | 0.82^***^ | 0.72^***^ | 0.77^***^ | 0.33^***^ | 0.80^***^ | -0.15^*^ |
| RF | 0.91^***^ | 0.26 | 0.88^***^ | 0.19^***^ | 0.92^***^ | -0.10 |
| GBM | 0.80^***^ | 0.75^***^ | 0.71^***^ | 0.34^***^ | 0.75^***^ | -0.10 |
| MaxEnt | 0.80^***^ | 0.65^***^ | 0.66^***^ | 0.30^***^ | 0.69^***^ | -0.10 |
| GAM | 0.78^***^ | 0.73^***^ | 0.70^***^ | 0.28^***^ | 0.78^***^ | -0.14 |
| GLM | 0.78^***^ | 0.69^***^ | 0.66^***^ | 0.27^***^ | 0.78^***^ | -0.16^*^ |
| ANN | 0.65^***^ | 0.65^***^ | 0.56^***^ | 0.25^***^ | 0.63^***^ | -0.13 |
| MARS | 0.77^***^ | 0.65^***^ | 0.68^***^ | 0.32^***^ | 0.81^***^ | -0.17^*^ |
| CTA | 0.76^***^ | 0.74^***^ | 0.68^***^ | 0.23^***^ | 0.80^***^ | -0.03 |
| SRE | 0.85^***^ | 0.13 | 0.79^***^ | 0.02 | 0.76^***^ | -0.21^**^ |

Reported values are the Spearman’s ρ coefficients of observed versus predicted range size in t_1_ (range size column) and observed versus predicted change in range size between time periods (range change column) for each modelling framework and major taxonomic group modelled. Stars indicate the significance level of correlations: ^*^ = p < 0.05; ^**^ = p < 0.01; ^***^ = p < 0.001.
