# Supplementary material for: Climatic Associations of British Species Distributions Show Good Transferability in Time but Low Predictive Accuracy for Range Change
Source: PLoS One. 2012 Jul 5;7(7):e40212. doi: 10.1371/journal.pone.0040212 (PMC3390350; doi:10.1371/journal.pone.0040212)
Supplement: Table S4 — Relative effect of taxonomic and methodological variation on accuracy of hindcasts. (DOCX) [file pone.0040212.s007.docx]

**Table S4. Relative effect of taxonomic and methodological variation on accuracy of hindcasts.**

|  | **AUC** | **Sensitivity** | **Specificity** | **CCR_stable_** | **CCR_changed_** |
| --- | --- | --- | --- | --- | --- |
| Species | 51.72 | 46.40 | 50.26 | 38.72 | 44.27 |
| Technique | 26.07 | 12.52 | 21.14 | 23.63 | 2.83 |
| Group | 1.88 | 1.81 | 1.54 | 2.52 | 3.23 |
| Residual | 20.33 | 39.26 | 27.06 | 35.13 | 49.68 |

The values reported are the results of a variance components analysis of the linear mixed-effects (LME) models investigating the factors affecting the accuracy of hindcasts. AUC, sensitivity, specificity of the entire range, as well as the correct classification rate of grid squares that have remained occupied or unoccupied (CCR_stable_), and the correct classification rate of grid squares that have changed occupancy status between time periods (CCR_changed_) were modelled as a function of the following random effects: modelling framework (n = 10), major taxonomic group (n = 3) and species (n = 1823). The ratio between the variance explained by each random effect and null variance (expressed as a percentage) is reported for each random effect in each model.
