# Supplementary material for: Climatic Associations of British Species Distributions Show Good Transferability in Time but Low Predictive Accuracy for Range Change
Source: PLoS One. 2012 Jul 5;7(7):e40212. doi: 10.1371/journal.pone.0040212 (PMC3390350; doi:10.1371/journal.pone.0040212)
Supplement: Appendix S1 — Description and optimisation of the modelling techniques used. (DOCX) [file pone.0040212.s008.docx]

**Appendix S1. Description and optimisation of the modelling techniques used.**

**Classification tree analysis** (CTA; [1]) is a classification technique attempting to explain variation in a single response variable by one or more explanatory variables by means of a tree. Trees are constructed by repeatedly splitting the data, following a set of decision rules based on the predictor variables. Each split is determined by a rule based on a single predictor variable and partitions the data into two mutually-exclusive groups. Splits for all of the predictors are examined by an exhaustive search procedure and the best split (i.e., the one maximizing the homogeneity of the two resulting groups with respect to the response variable) is chosen. The splitting procedure is then applied to each group separately. The objective is to partition the response into homogeneous groups, but also to keep the tree reasonably small. The procedure initially grows maximal trees and then uses techniques such as cross-validation (CV) to prune the over-fitted tree to an optimal size. In this analysis, CTA was carried out using a 10-fold cross-validation to select the best trade-off between the number of leaves on the tree and the explained deviance.

**Generalised linear models** (GLMs; [2]) are mathematical extensions of linear models. They relate a response variable to a set of predictors through a link function, which allows transformation to linearity without forcing the data into unnatural scales, thereby allowing for non-linearity and non-constant variance structures in the data. Here, GLMs were generated assuming a logistic link function and a binomial error distribution of the response. Linear, quadratic and polynomial terms (second and third order) of each climatic predictor were included in the initial models, and a stepwise procedure using the AIC criterion was used to select the most significant terms [3].

**Generalised additive models** (GAMs; [4]) are semi-parametric extensions of GLMs, which allow predictors to be modelled non-parametrically. They use a link function to establish a relationship between the mean of the response variable and a ‘smoothed’ function of the predictors. Here, GAMs were generated with cubic-smooth splines bounded by a degree of smoothness of four for each climatic predictor. A stepwise procedure similar to GLMs was used to select the most parsimonious models.

**Multivariate adaptive regression splines** (MARS; [5]) are non-parametric regression procedures in which non-linear responses between a species and a predictor are described by a series of linear segments of differing slope – each of which is fitted using a basis function – and knots – which define breaks between segments – which are entirely estimated from the data. In other words, a non-linear MARS function consists of a series of connected straight line segments, rather than the smooth curve of a GAM. Here, MARS were fitted with two-level interactions between predictors.

**Feed-forward artificial neural networks** (ANNs; [6]) are non-linear statistical modelling tools simulating the structure and the functional aspects of biological neural networks. ANNs consist of three types of inter-connected neuron layers (an input layer, one or more hidden layers, and an output layer), and use a back-propagation algorithm to feed the output of a layer forward to the next layer. A modifiable weight is associated to each connection between two successive layers of neurons, which is a function of the sum of the inputs to the node modified by a simple nonlinear transfer function. Such systems can approximate complex non-linear functions thanks to their ability to learn through training. During training, ANNs are repeatedly presented with the training data and the weights in the network are adjusted until the calculated output for a given input vector is as close as possible to the desired output. The accuracy of ANNs is mainly controlled by two parameters: the amount of weight decay and the number of hidden units. In this analysis, these two parameters were set to 0.03 and 7, respectively. Due to their heuristic nature, ANNs were run 10 times for each species and the average prediction used.

**Generalised Boosted Models** (GBMs; [7]) are sequential, stage-wise procedures which involve fitting multiple trees iteratively to the training data, and then merging their results together. At each stage, a tree is fitted to the residuals of the preceding tree, in an attempt to increase the modelling accuracy of those observations poorly modelled by the existing collection of trees. Fitted values in the final model are computed as the sum of all trees multiplied by the learning rate (i.e. the desired contribution of each tree to the final model). The model-building process performs best if it moves slowly down the gradient, so the contribution of each tree is usually shrunk by a learning rate that is substantially less than one. In this analysis, GBMs were fitted with an interaction depth of 4, a learning rate of 0.001, and a maximum of 5000 trees fitted to the data.

**Random Forests** (RFs; [8]) are, similarly to GBMs, a proposed improvement on single classification trees. This technique combines the principle of bagging trees with the random selection of features. In RF, multiple trees are generated from several datasets created by re-sampling with replacement (i.e. bagging). In addition to this, each tree is grown with a randomized subset of predictors. Random predictor selection diminishes correlation among trees and keeps biases low. Multiple trees are grown to maximum size without pruning, and the fitted values in the final model are computed by averaging all trees. The predicted output depends on two user-selected parameters, the number of trees grown and the number of predictors to be chosen randomly at each node. In this analysis, these two parameters were set to 500 and (total number of predictors - 1), respectively.

**Surface range envelopes** (SREs; [9]*)* are a profile technique similar to BioClim (Busby 1991), which generates a *p*-dimensional rectilinear environmental envelope for each species, based on the maximum and minimum values of the *p* environmental predictors from the set of locations where that species occurred. All environmental values for all locations in the study area are then reviewed by the model to check whether they fall inside or outside the species’ environmental envelope, generating binary predictions of the species’ potential range. For a more refined analysis, potential ranges can also be determined by identifying locations with climatic values contained within fractional parts of the total range of the species’ climate envelope. Here, absence locations were only included to the species’ potential range if falling within the 2.5- and 97.5-percentiles of the species’ environmental envelope.

**Maximum entropy** (MaxEnt; [10]) is a presence-only modelling technique which estimates a target probability distribution for each species by finding the probability distribution of maximum entropy (i.e., closest to uniform) subject to a set of constraints (environmental variables). We enabled the use of all six feature classes (linear, product, quadratic, hinge, threshold and categorical) for modelling species responses to environmental variables. The default value of 1.0 was used as the regularization parameter, which affects how closely the training data is fitted.

**REFERENCES**

1. Breiman L, Friedman J, Stone CJ, Olshen RA (1984) Classification and regression trees. London: Chapman and Hall. p.

2. McCullagh P, Nelder JA (1989) Generalized linear models. London: Chapman and Hall. p.

3. Akaike H (1974) A new look at statistical model identification. IEEE Transactions on Automatic Control AU-19: 716–722.

4. Hastie T, Tibshirani R (1990) Generalized additive models. London: Chapman and Hall. p.

5. Friedman JH (1991) Multivariate adaptive regression splines. The Annals of Statistics 19: 1–67.

6. Ripley B (1996) Pattern recognition and neural networks. Cambridge, UK: Cambridge University Press. p.

7. Ridgeway G (1999) The state of boosting. Computing Science and Statistics 31: 172–181.

8. Breiman L (2001) Random forests. Machine Learning 45: 5–32.

9. Busby J (1991) BIOCLIM – a bioclimate analysis and prediction system. In: Margules CR, Austin MP, editors. Nature conservation: cost effective biological surveys and data analysis. Canberra, Australia: CSIRO. pp. 66–68.

10. Phillips SJ, Anderson RP, Schapire RE (2006) Maximum entropy modeling of species geographic distributions. Ecological Modelling 190: 231–259.
